# Long-term stability and protection efficacy of the RBD-targeting COVID-19 mRNA vaccine in nonhuman primates

**DOI:** 10.1038/s41392-021-00861-4

**Published:** 2021-12-24

**Authors:** Hui Zhao, Tie-Cheng Wang, Xiao-Feng Li, Na-Na Zhang, Liang Li, Chao Zhou, Yong-Qiang Deng, Tian-Shu Cao, Guan Yang, Rui-Ting Li, Yi-Jiao Huang, Yuan-Guo Li, Yi-Ming Zhang, Fang-Xu Li, Yu-Ren Zhou, Yu-Hang Jiang, Xi-Shan Lu, Shi-Hui Sun, Meng-Li Cheng, Kai-Ping Gu, Mei Zhang, Qing-Qing Ma, Xiao Yang, Bo Ying, Yu-Wei Gao, Cheng-Feng Qin

**Affiliations:** 1 State Key Laboratory of Pathogen and Biosecurity, Beijing Institute of Microbiology and Epidemiology, Academy of Military Medical Sciences, 100071 Beijing, China; 2 Key Laboratory of Jilin Province for Zoonosis Prevention and Control, Changchun, 130122 China; 3 School of Medicine, Tsinghua University, 100084 Beijing, China; 4 State Key Laboratory of Proteomics, Beijing Proteome Research Center, National Center for Protein Sciences (Beijing), Beijing Institute of Lifeomics, 102206 Beijing, China; 5 Shandong Normal University, Jinan, 250014 China; 6 Suzhou Abogen Biosciences, Suzhou, 215123 China; 7 Research Unit of Discovery and Tracing of Natural Focus Diseases, Chinese Academy of Medical Sciences, 100071 Beijing, China

**Keywords:** Vaccines, Vaccines

## Abstract

Messenger RNA (mRNA) vaccine technology has shown its power in preventing the ongoing COVID-19 pandemic. Two mRNA vaccines targeting the full-length S protein of SARS-CoV-2 have been authorized for emergency use. Recently, we have developed a lipid nanoparticle-encapsulated mRNA (mRNA-LNP) encoding the receptor-binding domain (RBD) of SARS-CoV-2 (termed ARCoV), which confers complete protection in mouse model. Herein, we further characterized the protection efficacy of ARCoV in nonhuman primates and the long-term stability under normal refrigerator temperature. Intramuscular immunization of two doses of ARCoV elicited robust neutralizing antibodies as well as cellular response against SARS-CoV-2 in cynomolgus macaques. More importantly, ARCoV vaccination in macaques significantly protected animals from acute lung lesions caused by SARS-CoV-2, and viral replication in lungs and secretion in nasal swabs were completely cleared in all animals immunized with low or high doses of ARCoV. No evidence of antibody-dependent enhancement of infection was observed throughout the study. Finally, extensive stability assays showed that ARCoV can be stored at 2–8 °C for at least 6 months without decrease of immunogenicity. All these promising results strongly support the ongoing clinical trial.

## Introduction

The coronavirus disease 2019 (COVID-19) caused by severe acute respiratory syndrome coronavirus 2 (SARS-CoV-2) has aroused global concern. The pandemic of COVID-19 has caused more than 211 million confirmed cases and over 4.43 million deaths all over the world till 23 August, 2021 (WHO, 2021, https://covid19.who.int). Some countries like China have managed to contain the epidemic of SARS-CoV-2 through rigorous public health interventions such as the use of face mask.^1,2^ However, developing safe and effective vaccines and building herd immunity are the key to end the pandemic.

SARS-CoV-2 is a typical positive-sense, single-stranded RNA virus, which belongs to the genus *Betacoronavirus* (β-CoV) of the family *Coronavirdae*. The genome of SARS-CoV-2 encodes four structural proteins including spike (S), envelope (E), membrane (M), and nucleocapsid (N), 16 nonstructural proteins (nsp1-nsp16) and several accessory proteins.^3^ SARS-CoV-2 enters human cells mediated by the interaction between host angiotensin-converting enzyme 2 (ACE2) and the receptor-binding domain (RBD) of viral S protein.^4^ The full-length S protein, as well as RBD, are fully capable of inducing protective neutralizing antibodies (NAbs) and cellular immunity.^5^ Immunization with mRNA vaccine encoding the SARS-CoV-2 RBD was demonstrated to elicit robust NAbs and memory T, B cell responses in rodents without the detection of antibody-dependent enhancement (ADE) of infection.^6,7^

More than 130 vaccine candidates against SARS-CoV-2 are in clinical development, and at least 55 of them have reached phase III clinical trials as of Nov 2021(WHO, 2021, https://www.who.int/publications/m/item/draft-landscape-of-covid-19-candidate-vaccines). Among them, messenger RNA (mRNA) vaccines represent as a powerful and universal platform technology due to its ability for rapid development, high potency, and potential for large-scale production. A panel of mRNA vaccine candidates against viral diseases, e.g., influenza, rabies and acquired immune deficiency syndrome (AIDS), have been developed with ideal safety and immunogenicity in preclinical studies and clinical trials.^8–10^ Recently, two mRNA vaccines against COVID-19, one from Pfizer-BioNTech and the other from Moderna, have been well demonstrated with ideal safety and efficacy profiles in clinical trials^11,12^ and real-world studies.^13^

Recently, we have developed a novel COVID-19 mRNA vaccine candidate, ARCoV, based on the established lipid nanoparticles (LNPs) encapsulated mRNA platform.^14^ ARCoV was designed to carry mRNA encoding the RBD of SARS-CoV-2, and two doses of ARCoV vaccination conferred full protection against SARS-CoV-2 challenge in murine models.^14^ However, the protection efficacy in nonhuman primates remains not investigated. Additionally, mRNA vaccines need to be stored and transported at very low temperatures,^15^ which significantly limited the accessibility in developing and resource-limited countries. Our previous data showed the formulation of ARCoV maintained stable after storage at 2–8 °C for at least 1 month.^14^ However, the long-term stability profile of ARCoV remains to be determined. Herein, we evaluated the protection efficacy of ARCoV against SARS-CoV-2 challenge in an established cynomolgus macaque model, and determined the long-term stability of ARCoV at refrigerator temperature. The encouraging results reported here support further clinical development of ARCoV.

## Results

### Humoral and cellular immune responses in ARCoV-immunized macaques

**Fig. 1:**
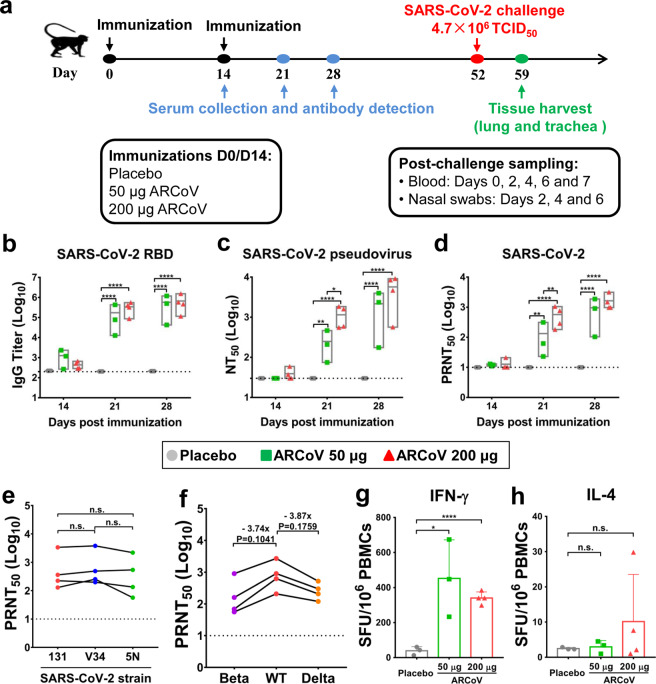
Humoral and cellular immune responses in ARCoV-immunized cynomolgus macaques. Cynomolgus macaques were immunized i.m. with 50 μg (*n* = 3) or 200 μg (*n* = 4) of ARCoV or a Placebo (*n* = 3) and boosted with the same dose on day 14 after first vaccination. **a** Schematic diagram of vaccination, sampling, and viral challenge in cynomolgus macaques. **b** SARS-CoV-2 RBD-specific IgG antibody titers of the immunized animals were determined by ELISA. **c**, **d** The NT_50_ and PRNT_50_ titers of the immunized animals were detected using VSV-based pseudovirus and live SARS-CoV-2, respectively. The dashed lines indicate the detection limit of the assay. Data are shown as mean with floating bars (min to max). Symbols represent individual animals. Statistical significance was calculated using a Student’s *t* test (n.s. not significant; *p < 0.05, **p < 0.01, ****p < 0.0001). **e**, **f** Serum cross-neutralization assays against SARS-CoV-2 epidemic strains and variants of concern in animals immunized with 200 μg of ARCoV. PRNT_50_ were performed using animal sera collected on day 28 after the first vaccination. The data were analyzed by a Paired *t*-test. **g**, **h** ELISpot assays for IFN-γ and IL-4 in PBMCs of the ARCoV-immunized cynomolgus macaques. Data are shown as mean ± SEM. Significance was calculated using a Student’s *t* test (*p < 0.05, ****p < 0.0001)

To characterize the immunogenicity of ARCoV in nonhuman primates, groups of cynomolgus macaques were immunized with 50 or 200 μg of ARCoV, respectively. Placebo group animals were immunized with 200 μg of empty LNPs. All animals were boosted with the same dose of ARCoV or Placebo on day 14 post the primary vaccination, and sera were collected on day 14, 21 and 28 post the first vaccination and subjected to SARS-CoV-2-specific antibody assays (Fig. 1a). The SARS-CoV-2-specific IgG antibody levels in animals immunized with 50 or 200 μg of ARCoV increased in a dose-dependent manner after the boost vaccination, reaching 576,316 and 670,750 on day 28 after vaccination, respectively (Fig. 1b). Similarly, there was a dose-dependent increase in neutralizing antibody titers measured with an established VSV-based pseudovirus system.^16^ Animals vaccinated with 200 μg of ARCoV had a 50% neutralization tests (NT_50_) of 37 on day 14 after the first vaccination, which increased to 3777 on day 28 post initial vaccination; the NT_50_ in animals vaccinated at the lower dose approached 1179 on day 28 post initial vaccination (Fig. 1c). More importantly, standard 50% plaque reduction neutralization tests (PRNT_50_) with live SARS-CoV-2 showed the neutralization antibody titers in animals vaccinated with 50 or 200 μg of ARCoV readily increased to 560 and 1446, respectively, after the boost vaccination (Fig. 1d).

Furthermore, to assay the cross-neutralization capability of ARCoV, two additional early epidemic strains, 5 N and V34, were used for PRNT_50_ assays with sera from animals immunized with 200 μg of ARCoV. The results showed that there was no significant difference among the PRNT_50_ titers against the three strains (Fig. 1e). Additionally, we also determined cross-neutralization capability against the newly defined variants of concerns, beta and delta variants. PRNT_50_ showed that sera from animals immunized with 200 μg of ARCoV were full capable of neutralizing beta and delta variants, and the PRNT_50_ titers were calculated to 296 and 286, respectively, which is 3.74-fold and 3.87-fold reduction in comparison with the wild-type SARS-CoV-2 (WT) (Fig. 1f).

We further profiled the SARS-CoV-2-specific T cell immune responses in ARCoV-immunized macaques. Enzyme-linked immunosorbent spot (ELISpot) assays showed that secretion of interferon γ (IFN-γ) in peripheral blood mononuclear cells (PBMCs) from animals immunized with ARCoV was significantly higher than that from animals received placebo vaccination (Fig. 1g). Meanwhile, no significant difference in IL-4 secretion were observed between ARCoV-immunized animals and Placebo-immunized ones (Fig. 1h). Together, these data demonstrated that ARCoV is full capable of inducing humoral and Th1-biased cellular immune responses against SARS-CoV-2 in cynomolgus macaques.

### Protective efficacy of ARCoV against SARS-CoV-2 challenge

**Fig. 2:**
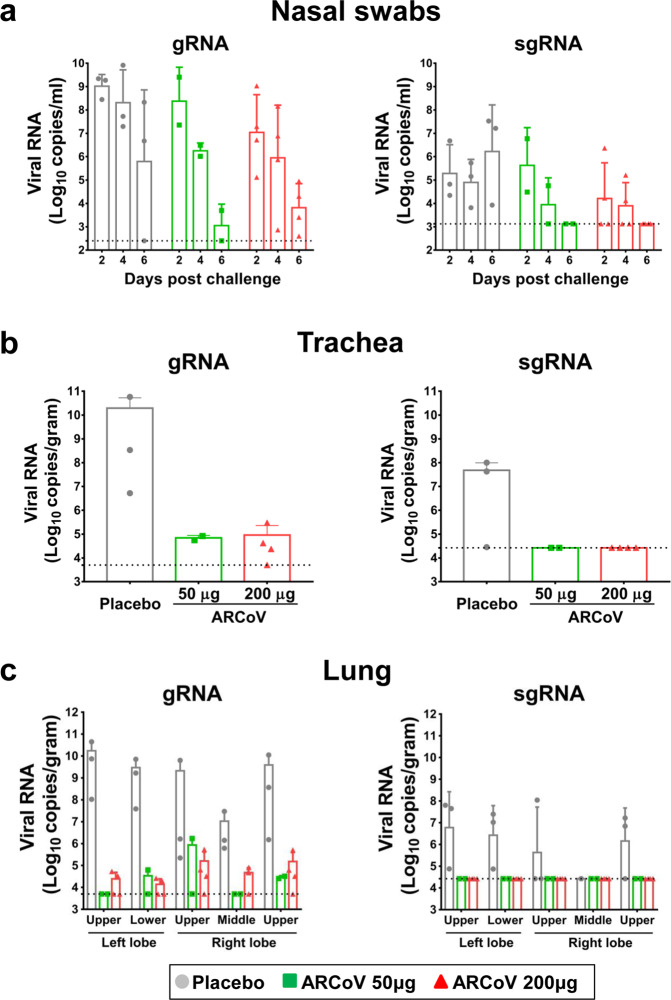
ARCoV vaccination prevents SARS-CoV-2 replication and secretion in cynomolgus macaques. Nasal swab (**a**) specimens were obtained on day 2, 4, and 6 after challenge. Trachea (**b**) and lung (**c**) specimens were obtained on day 7 after challenge. Viral load was assessed by analysis of SARS-CoV-2 genomic RNA (gRNA) and subgenomic RNA (sgRNA). Data are shown as mean ± SEM. The dashed lines indicate the detection limit of this assay

To evaluate the protective efficacy of ARCoV in cynomolgus macaques, all immunized animals were challenged with 4.7 × 10^6^ TCID_50_ of SARS-CoV-2 on day 52 after the first vaccination (Fig. 1a). All animals were sacrificed on day 7 post challenge, and lung, trachea and nasal swab specimens were collected as indicated for subsequent analysis. Both genomic RNA (gRNA)- and subgenomic RNA (sgRNA)- targeted RT-qPCR assays were performed to determine the viral RNA loads. In nasal swab specimens from the Placebo-immunized animals, high levels of viral gRNA and sgRNA were detected on day 2, 4 and 6 post challenge (Fig. 2a). In contrast, there was an obvious decrease trend of both gRNA and sgRNA loads from day 2 to day 6 post challenge in animals that received either 50 or 200 μg of ARCoV. More importantly, no viral sgRNA, indicative of infectious viruses,^17^ was detected in nasal swab specimens from all ARCoV-vaccinated animals on day 6 post challenge. This result demonstrates ARCoV vaccination successfully prevented viral secretion in upper respiratory tract of macaques.

As expected, high levels of viral gRNA and sgRNA were detected in trachea and individual lung lobe from most animals that received Placebo vaccination on day 7 post challenge (Fig. 2b, c). However, the viral gRNA loads in either trachea or individual lung lobe from ARCoV-vaccinated animals were undetectable or much lower than that from the Placebo group. Strikingly, no measurable sgRNA was detected in any trachea and lung lobe from macaques that received either 50 or 200 μg of ARCoV, indicating the absence of infectious viruses.

**Fig. 3:**
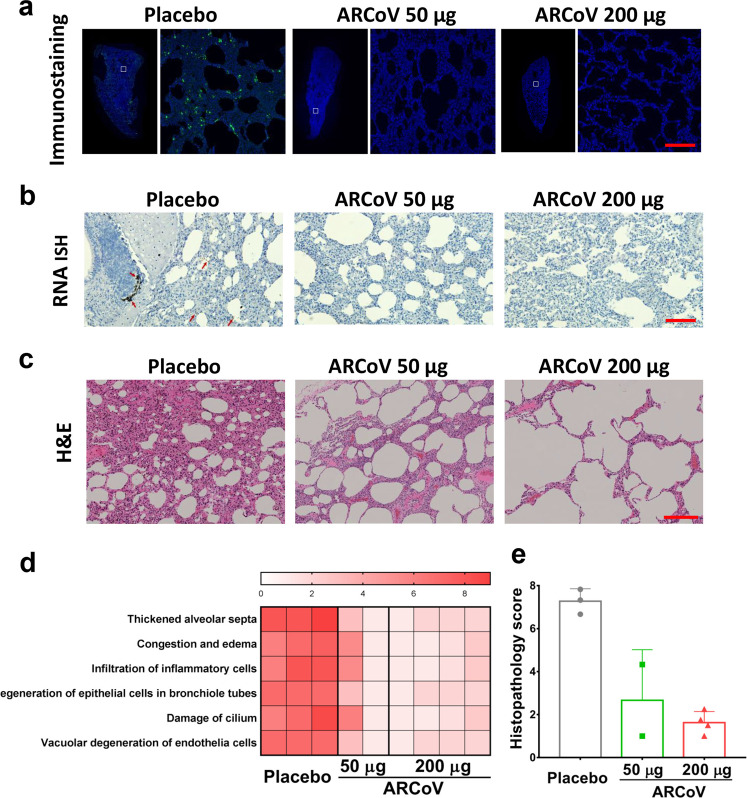
ARCoV vaccination protects from SARS-CoV-2 infection and its associated lung damages in cynomolgus macaques. **a** Immunostaining of lung tissues. SARS-CoV S proteins were indicated in green, and the area in the white box is magnified in right. Scale bar, 200 μm. **b** ISH assays for SARS-CoV-2 RNA. Positive signals are shown in brown (red arrowhead). Scale bar, 200 μm. **c** Hematoxylin and eosin (H&E) staining of lung tissues. Scale bar, 200 μm. **d** Heat map showing scores in each lung histopathological criterion from all animals. Severity in lung tissue lesions ranges from 0 to 9: 0 for normal, 1–3 for minimal, 4–6 for mild, 7–9 for moderate. **e** Graph for showing the average scores for all the lung histopathology criterions from the combined lobes per animal

Lung sections from all sacrificed animals were then subjected to immunostaining, in situ hybridization (ISH) and histopathological assays. Immunostaining assays showed abundant viral proteins were detected, mainly along the airway, in lung section from the Placebo animals, while few positive cells were detected in lungs from the ARCoV-vaccinated animals (Fig. 3a). Similarly, ISH assays showed obvious SARS-CoV-2-specific RNAs in lung sections from Placebo group, and only marginal viral RNA was detectable in all ARCoV-vaccinated animals (Fig. 3b). These results indicate that ARCoV was highly effective in eliminating SARS-CoV-2 replication in both upper and lower respiratory tracts of macaques.

More importantly, animals in the Placebo group developed typical interstitial pneumonia characterized by thickened alveolar septa, congestion and edema, accompanied by inflammatory cell infiltration, degeneration of epithelial cells in bronchiole tubes and damage of cilium and vacuolar degeneration of endothelia cells. In contrast, all animals vaccinated with ARCoV showed no obvious interstitial pneumonia, only mild histopathological changes were observed in lung sections (Fig. 3c). Further evaluation of lung lesions based on semiquantitative scoring system^18^ showed a significant reduction in severity of lung lesions in ARCoV-vaccinated animals compared to Placebo group, and the higher dose of ARCoV immunization conferred more beneficial effect in lung lesions (Fig. 3d, e). All these results clearly demonstrate that two doses of ARCoV vaccination successfully protected cynomolgus macaques from pulmonary lesions caused by SARS-CoV-2.

Additionally, we also assayed the serum cytokines and chemokines in response to SARS-CoV-2 challenge in all animals. As shown in Supplementary Fig. S1, SARS-CoV-2 challenge resulted in the up-regulation of large numbers of cytokines and chemokines, including IL-13, IL-7, MIP-1 alpha (CCL3), MIP-1 beta (CCL4), IL-6, stem cell factor (SCF), and MCP-1 (CCL2) in the Placebo group. In contrast, no change or down-regulation was detected in most cytokines in the ARCoV group. Especially, a significant down-regulation of MIP-1 beta and SCF were observed in either 50 or 200 μg of ARCoV group. These data show that inflammatory cytokines stimulated by SARS-CoV-2 was largely eliminated by ARCoV vaccination.

### The long-term stability of ARCoV at refrigerator temperature

**Fig. 4:**
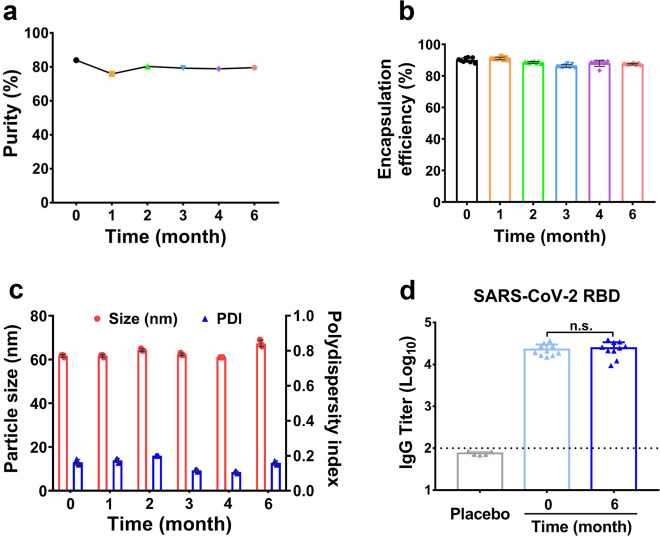
The long-term stability of ARCoV formulations. After storage for 0, 1, 2, 3, 4, and 6 months at 2–8 °C, all formulations were tested for mRNA purity (**a**), mRNA encapsulation efficiency (**b**), particle size and polydispersity index (PDI) (**c**). Data is represented as mean ± SEM (n = 8) for encapsulation efficiency, and (n = 3) for particle size and PDI. **d** Mice were immunized i.m. with two doses of Placebo (n = 5), fresh ARCoV (n = 10) or stored ARCoV (n = 10), with an interval of 14 days. The SARS-CoV-2 RBD-specific IgG antibody titers of the immunized mice on day 21 after initial vaccination was determined by ELISA. Symbols represent individual animals. Data are shown as mean ± SEM. The dashed lines indicate the limit of detection. Statistical significance was calculated using a Student’s *t* test (n.s. not significant)

The stability of ARCoV was evaluated using clinical batches manufactured under GMP conditions. After storage for 0, 1, 2, 3, 4, and 6 months at 2–8 °C, all formulations were tested for mRNA purity, mRNA encapsulation efficiency, particle size, and size distribution. The mRNA purity after 6 months storage maintained above 75.9% (Fig. 4a), and RNA quantitation assays showed the encapsulation efficiency maintained over 86% after 6 months storage (Fig. 4b). As shown in Fig. 4c, dynamic light scattering assays showed the particle size of ARCoV after storage ranged between 60 and 80 nm, and the polydispersity index (PDI) was less than 0.2. Finally, the immunogenicity of ARCoV after 6-month storage at 2–8 °C were determined in mice. Female BALB/c mice (6–8-week-old) were intramuscularly immunized with two doses of fresh or stored ARCoV as previously described.^14^ SARS-CoV-2 RBD-specific IgG antibody assays showed there was no significant between fresh and stored ARCoV (Fig. 4d). These physicochemical and biological properties of ARCoV after long-term storage at normal refrigerator temperature highlight an ideal stability profile of ARCoV.

## Discussion

ARCoV is an LNP-encapsulated mRNA vaccine, which is manufactured by a similar process as mRNA-1273 and BNT162b2. Previously, we have demonstrated that protective efficacy of ARCoV in the mouse model.^14^ In the present study, we demonstrated that protective efficacy of ARCoV in a nonhuman primate model. Two doses of ARCoV vaccination induced high level of neutralizing titers in a dose-dependent manner, and the NAbs titers in macaques immunized with 50 or 200 μg of ARCoV approached to 560 and 1446 (Fig. 1d), respectively. Although the NAbs titers are determined by different methods with different virus strains, the NAbs titers induced by ARCoV is comparable to the two mRNA vaccine candidates, mRNA-1273^12^ or BNT162b2.^11^ A recent study indicated that PRNT_50_ titer of 1:30 was sufficient to reduce the risk of reinfection by 50%,^19^ thus the high NAbs titers in macaques probably predicts a high protection efficacy. As expected, our results showed that the secretion of IFN-γ, but not IL-4, was significantly induced by ARCoV immunization in macaques (Fig. 1g, h), which also supports a Th1-prone T cell response.

Currently, SARS-CoV-2 variants of concern were linked to higher transmissibility and the potential immune escape from medical countermeasures.^20^ NAbs of sera from individuals vaccinated with BNT162b2 were 4.9-fold reduced against Beta variants relative to the WT virus, and 5.8-fold reduced against Delta variants.^21^ Potency of mRNA-1273 vaccine induced neutralizing antibodies against Beta variants showed 12.4-fold reduction.^22^ In this study, we also evaluated cross-neutralization capability of ARCoV-vaccinated sera against Beta and Delta variants, and the results showed 3.74- and 3.87-fold reduction in NAbs titers compared with the WT virus (Fig. 1f). Cross-neutralization tests with sera from clinical trials are being underway.

Nonhuman primates are susceptible to SARS-CoV-2 infection,^18,23^ and have been well used to assay the protection efficacy of COVID-19 candidate vaccines.^24–28^ In our study, all cynomolgus macaques received Placebo treatment supported robust viral replication in trachea and lungs as well as viral secretion in nasal swabs (Fig. 2). Remarkably, no viral sgRNAs were detected in trachea and individual lung lobes in all ARCoV-vaccinated animals, and viral sgRNA secretions in nasal swabs were eliminated on day 6 post challenge. These results support that ARCoV is capable of preventing SARS-CoV-2 replication in lower respiratory tracts and viral secretion in upper respiratory tracts of macaques. Secretory IgA is thought to critical for the protection against SARS-CoV-2.^29^ Previously, mRNA-1273 was evidenced to induce SARS-CoV-2 S-specific IgA responses in bronchoalveolar-lavage fluid of macaques,^12^ whether ARCoV has a similar effect remains to be determined.

Recently, diffuse alveolar hemorrhage (DAH) has been recorded in immunocompromised patients infected with severe SARS-CoV-2 infection.^30^ However, we didn’t observe pneumorrhagia in our cynomolgus macaque model. The major pathological changes in lung tissues were inflammation, and mild erythrocyte diapedesis in the alveoli was accidentally observed. Consistently, DAH was also rarely observed in other nonhuman primates infected with SARS-CoV-2.^18,23^ The different pathological changes in animal models and human patients upon SARS-CoV-2 infection warrant further investigation.

Accumulating evidences indicated that a subgroup of severe COVID-19 patients might have a cytokine storm syndrome, characterized by increased IL-2, IFN-γ, IL-7, MIP-1, TNF-alpha, MCP-1, IL-6, etc.^31–33^ In agreement with the clinical findings, SARS-CoV-2 infection in cynomolgus macaques also resulted in the up-regulation of a large number of cytokines and chemokines (Supplementary Fig. S1). In contrast, inflammatory cytokine induction, especially IL-7, MIP-1 beta, and SCF, was limited in both ARCoV groups, which suggests that there was control of virus sufficient to limit excessive inflammatory responses. These results also support the no obvious inflammation was observed in the lungs of immunized nonhuman primates after challenge. Additionally, SCF is an inflammatory cytokine that is enhanced in many diseases,^34,35^ the correlation between SCF and COVID-19 warrants further investigation.

Another interesting point we would point out is that no evidence of ADE was observed through the study. All ARCoV-vaccinated animals showed no sign of enhanced viral replication or diseases. This is consistent with most previous findings and may partially attributed to the antigen used. The RBD antigen has never been shown to induce ADE of SARS-CoV-2 infection.^36,37^ A recent in vitro study showed that the infectivity-enhancing antibodies recognized a specific site on the N-terminal domain (NTD) of SARS-CoV-2 S protein.^38^ However, another study suggested that these infection-enhancing antibodies targeted NTD could facilitate virus infection in vitro, but increased lung inflammation can hardly occur in antibody-infused macaques.^39^

Stability has been well recognized as one of major challenges for mRNA vaccine development. Stability of mRNA vaccine formulations is determined by mRNA itself as well as the LNP delivery system.^15^ Extensive stability analysis showed that ARCoV can be stored at 2–8 °C for at least 6 months without obvious change in physicochemical properties and immunogenicity. Although mRNA-1273 is claimed be stored at 2–8 °C for 30 days, and BNT162b2 up to 5 days,^15^ the detail stability profile of both vaccines has not been published. The mRNA of ARCoV vaccine encodes sequence-optimized RBD region of SARS-CoV-2, and the length of mRNA is only about 1100 nt, which is much shorter than that from mRNA-1273 and BNT162b2. The sequence and structure of mRNA, as well as the specific modification, are critical for the mRNA stability,^40^ and the next-generation of mRNA with improvement stability are being designed and characterized. Besides, the process control is crucial to generate uniform LNPs with good biocompatibility, and the PDI value (<0.2) of different batches of ARCoV supports the stability described here.

Conclusively, we report the protection efficacy of the RBD-targeting mRNA vaccine in nonhuman primates, and provide evidences of long-term stability of ARCoV at 2–8 °C. These results are not only critical for further clinical development but also provide substantial novel informations to the field of mRNA vaccine. Overall, all these promising results support the ongoing clinical trials for ARCoV both in China and aboard, and a multi-regional phase 3 clinical trial has been launched recently.

## Materials and methods

### Ethics and biosafety statement

All animal procedures were reviewed and approved by institutional animal care and use committee of Laboratory Animal Center, Academy of Military Medical Sciences (AMMS) (Assurance Number: IACUC-DWZX-2020-030).

All vaccination experiments were performed in Laboratory Animal Center, AMMS in strict accordance with the guidelines set by the national standard of Laboratory animals and Laboratory animal-Requirements of environment and housing facilities. All challenge experiments were performed in animal biosafety level 3 (ABSL3) laboratory at the Key Laboratory of Jilin Province for Zoonosis Prevention and Control, Institute of Military Veterinary Medicine.

### Cells and viruses

African green monkey kidney cells (Vero) from American Type Culture Collection (ATCC), and human hepatocarcinoma cells (Huh7) from Japanese Cancer Research Resources Bank (JCRB) were cultured in Dulbecco’s minimal essential medium containing 10% fetal bovine serum (FBS), 100 U/mL penicillin, and 100 μg/mL streptomycin. All the cell culture reagents were purchased from Thermo Fisher Scientific, USA. SARS-CoV-2 strains, 131 (GWHACAX01000000), V34 (GWHACBB01000000), and 5N (GWHAMKA01000000), were isolated from COVID-19 patients in China early 2020. The two variants of concerns, beta (CSTR.16698.06.NPRC 2.062100001) and delta (CSTR.16698.06.NPRC 6.CCPM-B-V-049-2105-6), were isolated from the imported patients from South Africa and India, respectively. All the isolates were passaged in Vero cells and was titrated by plaque assays. The VSV-based SARS-CoV-2 pseudoviruses were propagated and titrated in Huh7 cells as previously described.^16^ All in vitro experiments involving live SARS-CoV-2 were conducted under biosafety level 3 (BSL3) laboratory in AMMS.

### ARCoV formulation

The ARCoV mRNA encoding a codon-optimized RBD region of SARS-CoV-2 were prepared in LNPs formulations as described previously.^14^ In brief, lipid mixture, including 1,2-distearoyl-sn-glycero-3-phosphocholine (DSPC), an ionizable lipid, PEG-lipid, and cholesterol, was dissolved in ethanol, and then mixed with mRNA solution in 20 mM citrate buffer (pH 4.0) at a ratio of 2:1 (aqueous: ethanol) in a T-mixer. Formulations were then concentrated to the required concentration through a tangential flow filtration (TFF) membrane with 100 kD, and stored at 2~8 °C through a 0.22 μm filter until use.

### Macaque vaccination

Cynomolgus macaques with similar age and body weight (2.3–6.3 kg) were selected. Ten healthy adult macaques were immunized with 50 μg (n = 3), 200 μg (n = 4) ARCoV or LNP (Placebo,  n = 3), and boosted with the same dose of the corresponding immunogen on day 14 after first vaccination, respectively. Blood was collected before the vaccination and on day 14, 21 and 28 after initial vaccination, and used to evaluate SARS-CoV-2 RBD-specific IgG and neutralizing antibody levels. On day 7 after the booster vaccination, plasma samples were collected from the immunized monkeys and tested for IFN-γ and IL-4 with ELISpot.

### Serum antibody measurements

The SARS-CoV-2 IgG ELISA (Quantitative) kit (Wantai, China) was used to quantify total SARS-CoV-2 RBD-specific IgG antibody in serum according to the production instructions.^14^ Briefly, all sera from the immunized animals were heated at 56 °C for 30 min. The inactivated serum in serial dilution was then added to a blocked 96-well plate pre-coated with the SARS-CoV-2 RBD antigen (50 μL/well) and incubated for 30 min at 37 °C. After rinsing 5 times, horseradish peroxidase (HRP) labeled goat anti-mouse IgG (1:5000) (ZSGB-BIO, China) was added and incubated at 37 °C for 30 min. A chromogen solution was added following 5 times of washing with PBS. After incubation at 37 °C for 5–15 min, the reaction was terminated by adding stop solution and read by single wavelength 450 nm using a microplate reader (Bio Tek, USA). ELISA titers were calculated according to the manufacturer’s instructions.

A VSV pseudovirus-based neutralization test was carried out to evaluate SARS-CoV-2-specific neutralizing antibodies as described previously.^14^ In short, a 3-fold continuously diluted serum (100 μL) was incubated with SARS-CoV-2 pseudovirus containing 650 TCID_50_ (50 μL) at 37 °C for 1 h, and then were inoculated with 2 × 10^4^ Huh7 cells (100 μL) at 37 °C for 24 h per well. Following the incubation, 150 μL supernatant was removed, and 100 μL luciferase substrate (Perkinelmer, USA) was added to each well. Then the microplate was placed in the dark for 2 min at room temperature. Luciferase activity was recorded using a GloMax^®^ 96 Microplate Luminometer (Promega, USA). The 50% neutralization titers (NT_50_) are determined as a 50% reduction in serum dilution of relative luminescent units (RLUs) compared to the viral control wells using a nonlinear regression analysis tool (GraphPad Prism 7.0, GraphPad Software, USA).

As previously mentioned, live virus neutralizing antibodies were evaluated using 50% plaque reduction neutralization test (PRNT_50_).^14^ Briefly, the serum was heat inactivated at 56 °C for 30 min, then five-fold serial dilutions of serum were mixed with the same volume of virus to produce a mixture of 200 PFU per milliliter. After incubation at 37 °C for 1 h, a mixture of 250 μL was added to the 24-well plate containing confluent monolayers of Vero cells. Following incubation at 37 °C for 1 h, the cells were covered with 1% low-melting agarose (Promega, USA). After 72 h of incubation at 37 °C, the cells were fixed with 4% formaldehyde, and the plaques were stained with 0.2% crystal violet. A reduction in plaque count of 50% was used as the neutralization titers.

### Enzyme-linked immunosorbent spot (ELISpot)

Cellular immune responses in PBMCs of animals were evaluated using either IFN-γ or IL-4 pre-coated ELISpot kits (MabTech, Sweden), according to the protocol.^14^ Briefly, the plates were blocked using RPMI 1640 medium containing 10% FBS and incubated for 30 min before plating cells. PBMCs of animals were cultured at 3 × 10^5^ cells/well, with SARS-CoV-2 RBD peptides (10 μg/ml), Concanavalin A or RPMI 1640 medium. Then plates were incubated at 37 °C, 5% CO_2_ for 36 h, and then were washed with TBST three times. The plates were incubated with biotinylated anti-monkey IFN-γ or IL-4 detection antibody at room temperature for 2 h, and then washed as described above. The plates were incubated for 1 h at room temperature with streptavidin-HRP. At last, AEC substrate solution was added to each well and the spots were counted using the automated EliSpot Reader Systems (AID, Germany). Assay results were expressed as (the number of spot in the experimental well-the number of spot in medium control)/10^6^ cells.

### Challenge experiments

On day 52 after the initial vaccination, all animals received a total dose of 4.7 × 10^6^ TCID_50_ of SARS-CoV-2 challenge. Viral solution was administered in 4 mL intratracheally, 0.5 mL intranasally (0.25 mL per nostril), and 0.2 mL ocularly (0.1 mL per eye), respectively. Sample collection before and after challenge is shown in Fig. 1a, and used for viral RNA measurement, immunofluorescence staining, ISH and histopathology assay.

### Detection of SARS-CoV-2 genomic RNA and subgenomic RNA

Blood and tissues were collected at different time points after the SARS-CoV-2 challenge. The tissues were weighed and homogenized, centrifuged at 8000 rpm for 10 min, and the supernatant was transferred to a new Ep tube. Viral RNA was extracted using the Magnetic Viral Nucleic Acid Kit (TIANGEN, China) in accordance with the manufacturer’s protocol. Viral genomic RNA (gRNA) quantification was carried out by Real-Time Quantitative Reverse Transcription PCR (qRT-PCR) targeting the ORF1ab and N gene of SARS-CoV-2 according to the information provided by the National Institute for Viral Disease Control and Prevention, China. As previously mentioned, subgenomic RNA (sgRNA) was used to quantify RNA replication in nasal swabs, lung, and trachea specimens.^17^ The limit of detection was the lowest level at which viral RNA can be specifically amplified in this assay (Ct value of 38).

### Immunofluorescence staining

The expression of SARS-CoV-2 S protein in lung sections was detected by immunostaining. The methods used are similar to those previously published.^14^ In brief, paraffin tissue sections were deparaffinized, rehydrated, and incubated with 3% H_2_O_2_ at room temperature. The sections were put in sodium citrate buffer (10 mM) for 1 h at 96 °C and blocked with BSA for 20 min. The primary antibody against SARS-CoV S protein (Sino Biologicals, China) was incubated at 37 °C for 2 h in a humidity control chamber, then the sections were detected using the TSA-dendron-fluorophores with NEON 7-color Allround Discovery Kit (Histova, China).

### In situ hybridization (ISH) assay

SARS-CoV-2-specific RNA in lung sections were determined by ISH assay as previously published.^14^ Briefly, 5 μm paraffin-embedded tissue sections were deparaffinized for 1 h at 60 °C. The endogenous peroxidases were quenched with hydrogen peroxide at room temperature for 10 min. Sections were boiled for 15 min in RNAscope Target Retrieval Reagents and incubated in RNAscope Protease Plus for 30 min before probe hybridization with RNAscope^®^ 2.5 HD Reagent Kit (Advanced Cell Diagnostics, USA). The tissues were counterstained with Gill’s hematoxylin and observed with a bright-field microscopy.

### Histopathology assay

For hematoxylin and eosin (H&E) histopathology evaluation, lungs were rapidly collected, and fixed in 4% formaldehyde for 48 h, followed by paraffin embedding. Serial sections with 4 µm thickness were prepared and selected sections (5–18 sections per animal) were stained with H&E for light microscopy examination. Images were captured using NIKON CI-S microscope equipped with a DS-FI2 camera. The lung tissue lesions were assessed according to the extent of thickened alveolar septa, congestion and edema, infiltration of inflammatory cells, degeneration of epithelial cells in bronchiole tubes, damage of cilium and vacuolar degeneration of endothelia cells. A semiquantitative scoring system was used to evaluate objectively the SARS-CoV-2-induced histopathological lesions. The degree of involvement in above each histopathological criterion was scored as: 0 for normal, 1–3 for minimal, 4–6 for mild, 7–9 for moderate. A maximum average score of 9 could be reached for the combined lobes per animal using this scoring system.

### Stability analysis of ARCoV

The mRNA purity was evaluated using 2100 Bioanalyzer (Agilent, USA) according to the manufacturer’s instruction. The mRNA encapsulation efficiency was measured using a Quant-iT™ RiboGreen™ RNA Assay Kit (Invitrogen, USA) according to the manufacturer’s instruction. Samples were excited at 480 nm and fluorescence intensity was measured at 520 nm, using a SpectraMax iD3 (Molecular Devices, USA). The measurements of each sample were taken 8 times. The LNP particle size and polydispersity index (PDI) of ARCoV were measured by dynamic light scattering (DLS) using a Zetasizer Nano-ZS (Malvern, UK). The measurements of each sample were taken 3 times. Results were analyzed using the Zetasizer software v7.13 (Malvern Panalytical, UK).

### Immunogenicity of ARCoV in mice

Female BALB/c mice (6–8-week-old) were immunized intramuscularly with fresh or stored ARCoV at 2–8 °C for 6 months (2 μg, n = 10), or Placebo (n = 5), respectively, and boosted with same dose of ARCoV on day 14 after first vaccination. Sera were collected on day 21 post primary vaccination to detect SARS-CoV-2 RBD-specific IgG antibodies.

### Statistical analysis

All data were analyzed with GraphPad Prism 7.0 software. Data are presented as mean ± SEM in all experiments. Student’s *t* test or Paired *t*-test was used to compare the means between two groups (*p < 0.05; **p < 0.01; ***p < 0.001; ****p < 0.0001; n.s. not significant).

## Supplementary information

Supplementary data

## Data Availability

All data collected in this study are available from the corresponding authors upon reasonable request.
