# Supplementary material for: Long-term stability and protection efficacy of the RBD-targeting COVID-19 mRNA vaccine in nonhuman primates
Source: Signal Transduct Target Ther. 2021 Dec 24;6:438. doi: 10.1038/s41392-021-00861-4 (PMC8703211; doi:10.1038/s41392-021-00861-4)
Supplement: Supplementary file 1 — Supplementary data [file 41392_2021_861_MOESM1_ESM.pdf]

Supplementary Materials for

Long-term stability and protection efficacy of the RBD-targeting COVID-19 mRNA vaccine in nonhuman primates

Hui Zhao, Tie-Cheng Wang, Xiao-Feng Li, Na-Na Zhang, Liang Li, Chao Zhou, Yong-Qiang Deng, Tian-Shu Cao, Guan Yang, Rui-Ting Li, Yi-Jiao Huang, Yuan-Guo Li, Yi-Ming Zhang, Fang-Xu Li, Yu-Ren Zhou, Yu-Hang Jiang, Xi-Shan Lu, Shi-Hui Sun, Meng-Li Cheng, Kai-Ping Gu, Mei Zhang, Qing-Qing Ma, Xiao Yang, Bo Ying, Yu-Wei Gao, Cheng-Feng Qin.

Correspondence to: Cheng-Feng Qin, qincf@bmi.ac.cn; Yu-wei Gao, yuwei0901@outlook.com; or Bo Ying, bo.ying@abogenbio.com

This PDF file includes:

Materials and Methods
Figure. S1

Materials and Methods

Cytokine secretion assays

Serum samples of animals on day 0 and 7 post challenge were collected by centrifugation and heat inactivated at 56℃. Serum concentrations of cytokines were measured with a ProcartaPlex NHP Cytokine/Chemokine/GF 37 plex (Invitrogen, #EXP370-40045-901) according to the manufacturer's instruction. Assays were performed on a Luminex 200™ machine equipped with xPONENT Software (ThermoFisher). We calculated the fold change of the cytokine level on day 7 post challenge compared with day 0. The heatmap of cytokines change were constructed using the pheatmap package (https://cran.rstudio.com/web/packages/pheatmap/index.html). Statistical significance was calculated by Mann-Whitney non-parametric t test.

Figure. S1

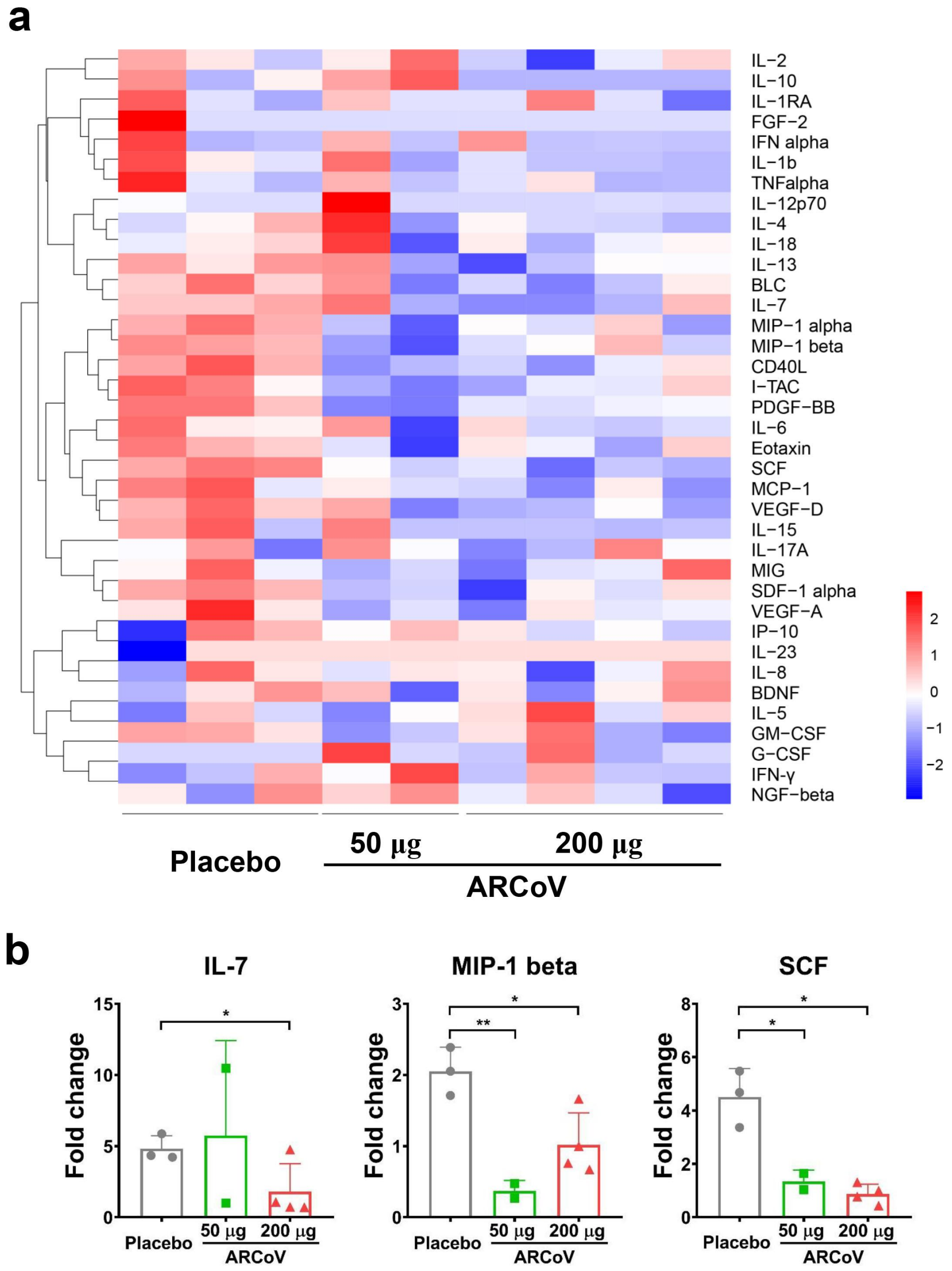

Fig. S1 Cytokines and chemokines in cynomolgus macaques challenged with SARS-CoV-2. a. Heatmap deciphering log2 transformed fold change in different groups. b. Statistical significance was calculated by Mann-Whitney non-parametric t test (*p< 0.05, **p< 0.01). The columns indicate median. Statistical P values for the different cytokines were: IL7, P = 0.049; MIP-1 beta, P = 0.0058 (50 μg ARCoV) and P = 0.018 (200 ug ARCoV); SCF, P = 0.023 (50 μg ARCoV) and P = 0.020 (200 μg ARCoV).
